# Recent Global Trends in Vaccinology, Advances and Challenges

**DOI:** 10.3390/vaccines11030520

**Published:** 2023-02-23

**Authors:** Amjad Islam Aqib, Aftab Ahmad Anjum, Md Atiqul Islam, Asad Murtaza, Aziz ur Rehman

**Affiliations:** 1 Department of Medicine, The Cholistan University of Veterinary and Animal Sciences, Bahawalpur 63100, Pakistan; 2 Quality Operation Labs Institute of Microbiology, University of Veterinary and Animal Sciences, Lahore 54000, Pakistan; 3 Centre for Immunology and Infection, Hong Kong Science and Technology Park, Pak Shek Kok, Hong Kong 999077, China; 4 School of Public Health, The University of Hong Kong, Hong Kong 999077, China; 5 Department of Pathobiology, University of Veterinary and Animal Sciences Lahore (Jhang Campus), Jhang 35020, Pakistan

## 1. Background

Vaccination/immunization plays a key role in maintaining the optimum health of humans and animals where the prevalence of disease is high and treatment becomes less effective. As antimicrobial resistance is rising day by day, the development of vac-cines becomes imperative to tackling emerging pathogens. According to the World Health Organization, vaccinations against cervical cancer, cholera, COVID-19, diphtheria, hepatitis B, influenza, Japanese encephalitis, malaria, measles, meningitis, mumps, pertussis, pneumonia, polio, rabies, rotavirus, rubella, tetanus, typhoid, varicella, and yellow fever in humans are being carried out in different countries depending upon the endemicity of the diseases. These account for vaccines against more than 20 life-threatening diseases that affect humans, and it has been observed that mere diphtheria, influenza, measles, and pertussis vaccines have prevented 3.5 to 5 million deaths annually. Vaccines against Ebola or malaria have yet to be made globally available; however, studies are being piloted. It was also found from experience that in the year 2021, more vaccines were introduced in a single year than ever before, and this was driven by COVID-19. Meanwhile, a tremendous decrease in vaccination since the year 2020 was noticed due to the COVID-19 pandemic, which deprived 25 million children of the opportunity to get vaccinated against various diseases in 2021. Immunization agenda 2030 (IA2030) is a goal to be achieved which intends to immunize everyone from all over the world in order to achieve good health and well-being [1]. Achieving IA2030 will save 24 million people possibly going into poverty by 2030 [2]. However, challenges continue to grow; e.g., climate change may lead to 60,000 additional deaths per year before 2050 because of malaria alone [1]. If we immunize three African countries against malaria, the pattern of this devastating trend may be changed [1]. On the other hand, as per reports from the World Health Organization for animals, devastating animal disease such as rabies, peste des petits ruminants (PPR), foot and mouth disease (FMD), anthrax, Aujeszky’s disease, bluetongue, brucellosis (Brucella abortus), Newcastle disease, avian influenza, lumpy skin disease, and brucellosis (Brucella melitensis) have both direct and indirect influences on public health and well-being in addition to the compromised health of animals [3,4]. Pathogenic strains are emerging in various parts of the world. As an example, subtypes of avian influenza have been reported in more than forty countries in the last six months. Hence, studies on vaccine efficacy are serious concerns in such situations. As some of the animal diseases are zoonotic and require extensive collaborative efforts, both animal and public health professionals are required.

## 2. Challenges

**Figure 1:**
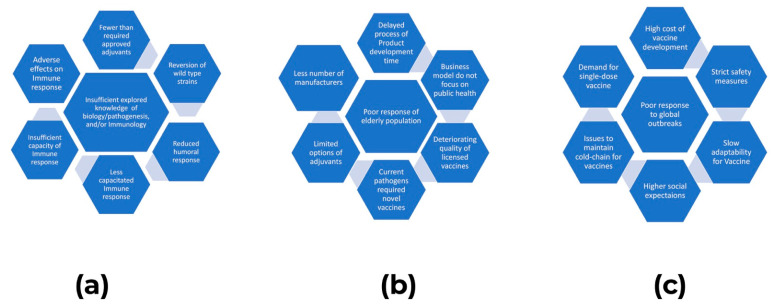
An overview (**a**–**c**) of the challenges in vaccine development and applications [5].

Conventional vaccines face challenges due to continued infections, extensive evolution in pathogens presenting high sequence variability, the complex of antigens, and emerging and re-emerging pathogens. There are salient barriers to the successful development of vaccines, including (1) incomplete understanding of how immunity develops, (2) host variability, (3) pathogen variability, (4) new vaccines and vaccine Safety, and (5) non-heritable factors [5]. Vaccines face a range of barriers that slow down the process of attaining their considerable benefits (Figure 1).

These issues are various; they include adjuvants, sustained efficacy of vaccines, adaptability of the public, knowledge gaps, reversal of virulence, biological factors at the end of recipient, loopholes in business models, quality maintenance, societal expectations, and many more. The extended challenges that will be faced in tackling emerging infectious diseases through immunization include, but are not limited to:

1. Poor health services in third world countries make it inaccessible for individuals to not only be immunized with new vaccines, but also to the basic, existing vaccines;
2. Safety is a major concern for some individuals that hinder them from getting vaccinated;
3. The process of developing a vaccine to launching it in the market takes years, and until that time, a particular disease may have already infected the majority of the population;
4. Genetic variability, especially in the RNA viruses, makes it challenging to make develop a vaccine against these pathogens;
5. Immunosenescence in elderly people hampers the effectiveness of vaccines, and elderly experience high morbidity and mortality from infections;
6. Some important pathogens, such as HIV, Malaria, and TB, evade and manipulate the host’s immune response, which makes it very difficult to develop a vaccine against these pathogens.

## 3. Approaches to Tackle Challenges (Adapted from [5,6])

- Studies on systems biology are required;
- Non-humoral correlates of protection are required to be identified;
- Effector functions that are related to quick recovery from infection should be identified and explored;
- Vaccines should have the ability to elicit a significant cellular response in addition to a humoral response, and vice versa;
- Population dynamics should be considered when producing and/or implementing vaccines;
- Vaccine response should be accurately measured from time to time, and for that, effective diagnostic tests should be used;
- Vaccines should produce a broad spectrum of neutralizing efficacies of antibodies;
- Multivalent vaccines should preferably be launched;
- Novel approaches to vaccine production, such as subunit vaccines, protein-based vaccines, and peptide-based vaccines with novel adjuvants, should be produced for durable and target-specific protection where applicable;
- Vaccines should employ dose-sparing approaches;
- Herd immunity, in the case of pandemics, is an effective solution;
- Impacts of aging on vaccines should be considered during their production;
- Safety should be a top priority;
- Business models should focus on public health at a priority;
- Access to the vaccines should be quick and unanimous for the public;
- Vaccine awareness should be equally prioritized with the same spirit by which the vaccine was produced, because anti-vaccine movements undo significant progress quickly;
- Genetic variabilities should be baseline for vaccine production;
- Novel approaches need to be explored to speed up the process of vaccine development. An example for this is COVID-19 vaccines, as with routine vaccine development, it takes 10 years for the vaccine to reach the public.

## 4. Advances in Vaccines

Nucleic acid vaccines, mRNA vaccines, vector vaccines, and biomaterial-based vaccines are recent trends in the revolutionization of vaccines based on their potential to address issues of existing technology. In addition to this, viral vectored vaccines (against Ebola virus), bacterial vectored vaccines, and antigen-presenting cells are more recent approaches. Employing nanotechnology, gene-based assays, next-generation sequencing technologies, and genetic engineering in the process of vaccine development is expected to enhance the revolutionary potential of novel vaccine types [7]. Other emerging, but more precisely relevant, technologies include the integration of multi-omics datasets, single-cell genomics, and epigenomic profiling of immunity, which have created new insights into the cellular and molecular responses that underly both the innate and adaptive immune system. It is also imperative to bring systems vaccinology into the loop in order to achieve our goals. System vaccinology uses metabolomics, transcriptomics, and mass cytometry, coupled with computational approaches, to prepare a global sketch of the complex mechanisms that occur during the development of immune responses against vaccination [8]. Two more important terms, vaccinomics and reverse vaccinology, are used for better outcomes. The former is used to study genomics and data at the system level to find the basis of variations at the individual level in the production of the immune response. The latter term explores genetic sequences to find immunogenic antigens [5,9].

## 5. Recommendations

It is crucial that we use different predictive models to accurately measure and predict the success of any running vaccine program or newly planned vaccine program, respectively. Failures of vaccine programs ought to be assessed at all tiers of animal health professionals, human health professionals, and professionals in environmental science. Hence, it is inevitable that these communities will work in collaboration with each other, adopting novel vaccines and vaccinology to achieve optimum health of animals and humans—promoting, in fact, one health.
